# *EGFR* plasma mutation in prediction models for resistance with EGFR TKI and survival of non-small cell lung cancer

**DOI:** 10.1186/s40169-019-0219-8

**Published:** 2019-01-19

**Authors:** Thang Thanh Phan, Bich-Thu Tran, Son Truong Nguyen, Toan Trong Ho, Hang Thuy Nguyen, Vu Thuong Le, Anh Tuan Le

**Affiliations:** 1 Biomolecular and Genetic Unit, Cho Ray Hospital, Ho Chi Minh City, 700000 Vietnam; 2 Faculty of Biology-Biotechnology, University of Science, VNU-HCM, Ho Chi Minh City, 700000 Vietnam; 3 Pathology Department, Cho Ray Hospital, Ho Chi Minh City, 700000 Vietnam; 4 Respirology Department, Cho Ray Hospital, Ho Chi Minh City, 700000 Vietnam; 5 Clinical Cancer Center, Cho Ray Hospital, Ho Chi Minh City, 700000 Vietnam

**Keywords:** *EGFR* plasma mutation test, Secondary T790M, cfDNA, NSCLC

## Abstract

**Background:**

This study aims to clarify the prognostic role of epidermal growth factor receptor (*EGFR*) mutations in plasma of non-small cell lung cancer (NSCLC) for resistance to tyrosine kinase inhibitor (TKI), in correlation with clinical characteristics. A total of 94 Adenocarcinoma, clinical stage IV NSCLC patients with either E19del or L858R mutation were admitted to the prospective study from Jan-2016 to Jul-2018. *EGFR* mutations in plasma were detected by scorpions ARMS method. The Kaplan–Meier and Cox regression methods were used to estimate and test the difference of progression-free survival (PFS) and overall survival (OS) between groups. The prognostic power of each factor was appraised by the Bayesian Model Averaging (BMA) method.

**Results:**

Among 94 patients, 28 cases still are good responses according to the RECIST criteria and negative for *EGFR* mutations in plasma. Of 66 resistant patients, *EGFR* mutations were positive in plasma of 57 cases (86.4%) which was higher than the value of pre-treatment (48.5%). Of which, 17 patients (25.8%) have the occurrence of *EGFR* mutations in plasma earlier than progression 2.1 (0.6–7.9) months. The secondary T790M mutation was found in the plasma of 32 cases (48.5%). Median PFS and OS for the study subjects were 12.9 (11.0–14.2) and 29.5 (25.2–41.3) months, respectively. The post-treatment *EGFR* plasma test with brain and new metastasis were detected as independent prognostic factors for worse PFS (*P *= 0.008, 0.016 and 0.028, respectively). While *EGFR* plasma (*P *= 0.044) with bone metastasis at baseline (*P *= 0.012), new metastasis (*P *= 0.003), and high cfDNA concentration (*P *= 0.004) serve as the worse survival factors, surgery treatment helps to prolong OS in NSCLC treated with EGFR TKI (*P *= 0.012). BMA analysis identified *EGFR* plasma test as the strongest prognostic factor for both PFS and OS (possibility of 100% and 99.7%, respectively).

**Conclusions:**

*EGFR* plasma test is the powerfully prognostic factor for early resistance with EGFR TKI and worse survival in NSCLC regardless of clinical characteristics.

## Background

Treatment with epidermal growth factor receptor (EGFR) tyrosine kinase inhibitor (TKI) such as Erlotinib or Gefitinib helps to prolong the progression-free survival (PFS) time, increase the response rate, and minimize the side effects compared to standard chemotherapy treatment for non-small cell lung cancer (NSCLC) patients who carried the activating *EGFR* mutations (deletions in exon 19—E19del, and L858R substitution mutation in exon 21) [1, 2]. Regrettably, most of that patients tend to be resistant with this TKI therapy after 9–15 months of treatment [3]. Intensive studies in the last decade have shown that various reasons were associated with acquired resistance to the first- and second-generation TKI, in which the secondary Threonine-790-Methionine substitution mutation (T790M) in exon 20 of the *EGFR* gene is the most common cause of TKI resistance (50–60%) [3].

Patients with this acquired mutation will be switched to the treatment with third-generation TKI such as Osimertinib which now is a standard therapy with higher efficiency compared to Erlotinib or Gefitinib, and chemotherapy [4, 5]. For monitoring the *EGFR* mutation status after treatment, especially the occurrence of the secondary T790M mutation, the tumor tissue samples from rebiopsy procedures are needed. However, it is difficult to perform biopsy repeatedly on the same patient. This procedure is not always successful [6, 7]. In these circumstances, the cell-free DNA (cfDNA) in plasma may serve as the surrogate sample with advantages of less invasion, fasting method, and convenient uses.

Previous studies have shown that *EGFR* mutation in general, and secondary T790M mutation can be detected in the plasma of resistant patients with high prevalence [8–21], and even be detected earlier than the disease progression [11, 14, 15, 21]. In the other context, the *EGFR* mutations in plasma also shown to be a prognostic factor for NSCLC patients treated with EGFR TKI, however with the contrary results. Some studies demonstrated that patients with positive-mutation in plasma at the baseline have a longer overall survival (OS) and progression-free survival (PFS) time compared to those of negative-result [22–25]. Contrariwise, other studies indicated that the De novo *EGFR* mutation in plasma is a poor prognostic factor for PFS and OS [21, 26, 27]. Whereas, the maintained positive status of *EGFR* mutation in plasma after treatment with EGFR TKI is also the worse factor for survival [13–15, 28, 29]. In the same way, the role of secondary plasma-T790M mutation in prognosis for NSCLC patients also was mentioned in previous studies with opposite opinions [9, 14, 15, 17, 19]. While the studies of Sueo-Aragane et al. [14], Zheng et al. [15], and Zhang et al. [17] shown that the occurrence of T790M in plasma after TKI treatment is the worse prognostic factor for OS and PFS, Sakai et al. [9] only noted this prognostic role of T790M in the patient group under 65 years old. Conversely, Wang et al. showed superior survival in the T790M positive group [19]. Moreover, most of these studies presented the prognostic role of *EGFR* plasma mutation in univariate analysis. Only studies of Zheng et al. and Wang et al. [15, 19] used the multivariate analysis, but with the different trend of prognosis. Our study aims to monitor the *EGFR* mutation status and the occurrence of T790M mutation in plasma of NSCLC patients after TKI treatment, subsequently, clarify the prognostic role of *EGFR* in general and secondary T790M mutation in correlation with clinical characteristics.

## Methods

### Patients and treatment evaluation

**Fig. 1:**
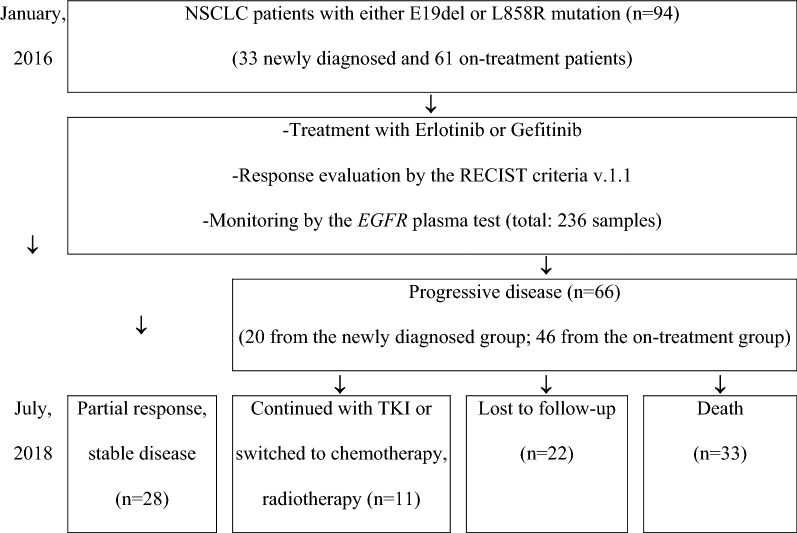
Patient groups and analysis flow

A total of 94 Adenocarcinoma, stage IV NSCLC patients (including 33 newly diagnosed cases) with either E19del or L858R mutation who were treated with Erlotinib or Gefitinib were selected for this study from January 2016 to July 2018 at Cho Ray hospital (approved by the Ethics Committees of Cho Ray hospital, reference number 602/2016 CN-HDDD). Patients were asked to participate in the study and informed in the consent form. Treatment evaluations were done every 2 months, based on the RECIST v1.1 criteria [30]. The progression-free survival was defined from the date of TKI treatment initiation to the date of first observation of progressive disease (PD). The overall survival was recorded as the time from disease diagnosis to death. By the end of July 2018, 28 patients had been being the stable disease (SD) or partial response (PR) with EGFR TKI, 66 patients developed clinical progression (20 cases from the newly diagnosed group and 46 cased from the on-treatment group) (Fig. 1). Of which resistant patients, 33 cases died after progression 1.3–21.5 months while 22 cases were lost to follow-up. The remained 11 resistant cases have been switched to chemotherapy, radiotherapy, or continued with EGFR TKI in combination with chemotherapy and/or radiotherapy.

### Sample collection and cfDNA extraction

The peripheral blood samples (5 mL, conserved in the EDTA tube) were collected at baseline (for 33 patients), every 2 months during the treatment process, and the progressive disease. Total of 236 blood samples from 94 patients was used for this study. Blood samples were centrifuged twice at 2000×*g*/4 °C/10 min and 12,000×*g*/4 °C/10 min to collect ~ 2 mL plasma. The cfDNA was extracted from 2 mL plasma by using kit QIAsymphony DSP Circulating DNA (Qiagen, Hilden, Germany) according to the instruction of the manufacturer. Briefly, 2 mL plasma was blended with 110 µL proteinase K, 110 µL magnetic particle solution, and 1780 µL biding buffer, then mixed by rod cover for 15 min. The particle-cfDNA complexes were selected by magnetic column and transferred to new wells and washed twice with QSW8 and QSW9 washing buffer, respectively. The cfDNA was eluted in 60 µL AVE buffer, and stored at − 80 °C until uses. All the above extraction steps were performed by automated QIAsymphony machine (Qiagen, Hilden, Germany).

### *EGFR* mutation detection and cfDNA quantification

*EGFR* mutations in plasma were detected by scorpions ARMS method, the Therascreen EGFR Plasma RGQ PCR kit, performed on the RotorGene Q 5Plex HRM platform (Qiagen, Hilden, Germany). Three *EGFR* mutation types assigned in this study were T790M (the acquired resistant mutation), E19del and L858R (the two activating mutations that are sensitive to EGFR TKI). The *EGFR* exon 2 was used in PCR reactions as reference gene (control reaction mix). Polymerase chain reactions (PCR) were prepared by mixing 5 µL cfDNA with 19.5 µL control reaction mix or mutation mix (E19del, T790M, L858R), and 0.5 µL Taq DNA polymerase. PCR temperatures were set up as follows: 95 °C/15 min; 40 cycles of 95 °C/30 s and 60 °C/60 s. The ΔCt value of each mutation type was calculated as mutation reaction Ct minus control reaction Ct. Patients were determined positive for *EGFR* mutations if ΔCt ≤ 8.00 (E19del), 8.90 (L858R) or 7.40 (T790M).

For cfDNA quantification, the human control DNA sample at 10 ng/µL (Qiagen, Germany) was used for dilution series of 10 ng/µL, 1 ng/µL, 0.1 ng/µL, and 0.01 ng/µL which were used in PCR assays with control reaction mix to build the standard curve. Parallel with each time of *EGFR* mutation testing, the concentration of cfDNA (ng/mL plasma) was reported.

### Statistical analysis

The Chi square or Fisher’s exact test, and Kruskal–Wallis rank test were used to compare the relative frequencies and cfDNA concentration between groups, respectively. The Kaplan–Meier statistical method was used to construct survival curves and calculate the median PFS and OS for *EGFR* mutation status, secondary T790M mutation status, and groups of clinical characteristics as the prognostic factors for resistance and death. The Cox regression model analysis with uni- and multivariate were used to compare the PFS and OS time between groups and calculate the hazard ratio (HR) with 95% confidence interval. Prognostic power of *EGFR* plasma mutation test and other clinical characteristics as confounders in the prediction model were assessed by the Bayesian Model Averaging (BMA) statistical method. All data analysis was performed on the R statistical software v.3.5.1 (R foundation, 1020 Vienna, Austria). *P *< 0.05 was considered as significant difference.

## Results

### Patient characteristics

Total of 94 Adenocarcinoma, clinical stage IV NSCLC patients with only E19del (61 cases, 64.9%) or L858R mutation (33 cases, 35.1%) at baseline had been enrolled in this study. Patients were categorized into two groups: the newly diagnosed group (n = 33) with available results of *EGFR* plasma test at baseline; and the on-treatment group (n = 61) who had been diagnosed before enrollment in the study. The characteristics of patients were presented in Table 1. The median age of all patients was 61 (32–89 years old). Among 94 cases, 53 cases (56.4%) were female and 41 cases (43.6%) were male patients.

**Table 1:** Patient characteristics

Characteristic	All patients (n = 94)	Newly diagnosed group (n = 33)	On-treatment group (n = 61)	*P* value
Age, years				0.949
Median (range)	61 (32–89)	60 (38–86)	61 (41–89)	
< 61	46	16	30	
≥ 61	48	17	31	
Gender				0.414
Female	53	17	36	
Male	41	16	25	
ECOG PS				0.986
0–1	77	27	50	
≥ 2	17	6	11	
Tumor location				0.896
Right lung	44	15	29	
Left lung	23	9	14	
Right + left	27	9	18	
Tumor size at baseline (cm)				0.914
≤ 3	17	6	11	
> 3–5	31	10	21	
> 5	46	17	29	
M-Staging				0.206
M0	13	4	9	
M1a	20	4	16	
M1b	61	25	36	
Brain metastasis at baseline				0.093
No	67	20	47	
Yes	27	13	14	
Bone metastasis at baseline				0.892
No	55	19	36	
Yes	39	14	25	
Liver metastasis at baseline				0.718
No	75	27	48	
Yes	19	6	13	
Pleural effusion at baseline				0.608
No	63	21	42	
Yes	31	12	19	
*EGFR* mutation type				0.791
E19del	61	22	39	
L858R	33	11	22	
*EGFR* plasma at baseline				–
Negative	17	17	–	
Positive	16	16	–	
Treatment method				0.750
TKI alone	55	21	34	
TKI + Che/Ra	17	5	12	
TKI + Sur	22	7	15	
Response				0.134
PR + SD	28	13	15	
PD	66	20	46	
Brain metastasis after treatment				0.986
No	77	27	50	
Yes	17	6	11	
Bone metastasis after treatment				0.207
No	62	19	43	
Yes	32	14	18	
Liver metastasis after treatment				0.277
No	69	22	47	
Yes	25	11	14	
Pleural effusion after treatment				0.131
No	68	27	41	
Yes	26	6	20	
New metastasis site				0.655
No	57	19	38	
Yes	37	14	23	
*EGFR* plasma after treatment				0.076
Negative	37	17	20	
Positive	57	16	41	
Secondary T790M mutation				0.308
Negative	62	24	38	
Positive	32	9	23	
Median cfDNA, ng/mL	183 (134–231)	175 (110–218)	199 (135–314)	0.276
≤ 200	54	22	32	
> 200	40	11	29	
Survival				0.173
Alive	39	16	23	
Dead	33	13	20	
Lost to follow-up	22	4	18	

*Che* chemotherapy, *PD* progressive disease, *PR* partial response, *Ra* radiotherapy, *SD* stable disease, *Sur* surgery

In clinical assessment, 77 cases (81.9%) were scored 0–1 with the criteria of Eastern Cooperative Oncology Group performance status (ECOG PS) while 17 cases (18.1%) with serious conditions were scored ≥ 2. Most of the patients have the tumors in right lung (71 cases, 75.5%) with tumor size larger than 3 cm (77 cases, 81.9%), and distant metastasis sites (M1b: 61 cases, 64.9%). The brain, bone, liver metastasis, and pleural effusion at baseline were recorded in 27 (28.7%), 39 (41.5%), 19 (20.2%) and 31 (32.9%) patients, respectively. Of 33 newly diagnosed patients, *EGFR* mutations were found in plasma samples of 16 cases (48.5%), including 13 cases with E19del mutation and 3 cases with the L858R mutation. The remaining 61 cases only have the mutation data (E19del or L858R) in tumor tissue samples at baseline. Until participation in the study, all of 61 cases had been being treated with Erlotinib or Gefitinib ≤ 6 months and archived good responses.

### Organ metastasis and *EGFR* mutation status after TKI treatment

Of 94 cases, 55 cases (58.5%) were treated with Erlotinib (150 mg/day) or Gefitinib (250 mg/day) alone while 22 cases (23.4%) were treated with Erlotinib or Gefitinib after surgical treatment with or without chemotherapy, and 17 cases (18.1) were treated with Erlotinib/Gefitinib after chemotherapy and/or radiotherapy (Table 1). By the end of July 2018, 28 cases still archived partial response or stable disease while 66 cases had developed clinical progression. The last records showed that brain, bone, liver metastasis, and pleural effusion occurred in 17 (18.1%), 32 (34.0%), 25 (26.6%) and 26 (27.6%) patients, respectively. New metastasis sites were observed in 37 (39.4%) patients.

Plasma of 28 patients with partial response or stable disease is still negative for *EGFR* mutations. Among 66 resistant patients, *EGFR* mutations were found in the plasma of 57 cases (86.4%) (16/20 resistant cases from the newly diagnosed group) which is higher than the data of pre-treatment (48.5%). The pooled mutation rate at post-treatment is 60.6% (57 of 94 patients). Mutations were more frequently detected in ≥ 61 years old (34/48, 70.8%) compared to < 61 years old group (23/46, 50.0%) (*P *= 0.039), in pre-positive patients (11/16, 68.7%) compared to pre-negative patients (5/17, 29.4%) (*P *= 0.014), and in patients developed bone metastasis after treatment (24/32, 75.0%) compared to remain group (33/62, 53.2%) (*P *= 0.041).

The secondary T790M mutation was found in 32 of 66 resistant patients, equivalent to the mutation rate of 48.5%. This data in the newly diagnosed group is 45.0% (9/20 resistant cases). Resistant patients with the E19del mutation have a higher rate of T790M (23/38, 60.5%) compared to patients with L858R mutation (9/28, 32.1%) (*P *= 0.023). Although with not statistically significant, the T790M mutation was observed more frequently in female patients (55.3%), in groups of baseline plasma positive for *EGFR* mutations (63.6%), bone metastasis (60.0%), and baseline liver metastasis (60.0%) compared to others.

Interestingly, we found that 17 of 66 resistant patients (25.8%) have the occurrence of *EGFR* mutations in plasma earlier than the progression time point. Of these 17 patients, 10 cases (58.8%) carried T790M mutation. The median time of early detection was 2.1 (ranging from 0.6 to 7.9) months.

The median cfDNA concentration in plasma of post-treatment was 183 (95% CI 134–231) ng/mL plasma. These values of resistant patients and PR + SD group were 199 (95% CI 154–295) and 115 (95% CI 75–215) ng/mL plasma, respectively (*P *= 0.009). Patients with positive mutations at post-treatment have the higher cfDNA concentration (196 ng/mL plasma) compared to others (126 ng/mL plasma) but not with significant difference (*P *= 0.094).

### Progression-free survival between groups and prognostic factors for resistance

The median PFS for all patients in this study was 12.9, 95% CI 11.0–14.2 months. The median PFS for sub-groups of *EGFR* mutation status, clinical characteristics and hazard ratios were estimated and shown in Table 2.

**Table 2:** Univariate analysis for progression-free survival

Characteristic	PFS, month (95% CI)	HR (95% CI)	*P*-value	HR^a^ (95% CI)	*P*-value^a^
Age, years		1.27 (0.78–2.09)	0.327	2.19 (0.97–5.94)	0.076
< 61	13.0 (11.3–16.0)				
≥ 61	12.0 (10.0–14.4)				
Gender		0.93 (0.58–1.37)	0.481	0.81 (0.63–1.98)	0.656
Female	12.0 (10.0–16.0)				
Male	12.9 (11.1–15.1)				
ECOG PS		1.65 (0.88–3.13)	0.119	1.74 (0.67–5.27)	0.336
0–1	13.0 (11.5–14.8)				
≥ 2	10.7 (7.9–16.1)				
Tumor location		0.87 (0.66–1.19)	0.351	0.79 (0.59–1.21)	0.195
Right lung	12.9 (10.4–15.1)				
Left lung	12.0 (8.8–23.5)				
Right + left	13.0 (9.5–16.6)				
Tumor size at baseline (cm)		1.07 (0.76–1.50)	0.685	1.05 (0.64–1.67)	0.855
≤ 3	13.0 (8.8–15.6)				
> 3–5	13.7 (11.3–19.3)				
> 5	12.6 (9.8–14.4)				
M-Staging		1.06 (0.75–1.50)	0.723	0.97 (0.58–1.59)	0.662
M0	9.7 (6.0–28.3)				
M1a	13.0 (8.8–19.7)				
M1b	12.6 (11.3–14.5)				
Brain metastasis at baseline		0.89 (0.56–1.53)	0.669	0.76 (0.45–1.72)	0.392
No	12.9 (10.4–14.8)				
Yes	12.6 (11.1–15.2)				
Bone metastasis at baseline		1.27 (0.78–2.08)	0.342	1.07 (0.62–2.65)	0.891
No	13.0 (10.0–17.5)				
Yes	12.3 (11.1–15.1)				
Liver metastasis at baseline		1.20 (0.77–2.15)	0.539	1.88 (0.81–5.72)	0.269
No	12.9 (10.0–14.2)				
Yes	11.5 (9.0–15.1)				
Pleural effusion at baseline		0.97 (0.57–1.64)	0.791	1.17 (0.67–2.86)	0.737
No	12.9 (11.1–14.8)				
Yes	12.6 (10.0–19.3)				
*EGFR* mutation type		1.45 (0.88–2.39)	0.139	1.84 (0.72–4.72)	0.204
E19del	13.7 (11.3–16.0)				
L858R	12.0 (9.5–13.8)				
*EGFR* plasma at baseline		1.43 (0.77–3.61)	0.346	1.22 (0.69–3.01)	0.672
Negative	12.0 (5.3-NR)				
Positive	9.7 (7.1–14.1)				
Unknown	13.0 (11.3–15.1)				
Treatment method		1.35 (0.99–1.83)	0.051	1.40 (0.81–2.44)	0.237
TKI alone	12.1 (9.8–14.2)				
TKI + Che/Ra	11.1 (9.0–13.0)				
TKI + Sur	14.8 (11.5–18.2)				
Brain metastasis after treatment		2.38 (1.30–4.38)	0.005	1.26 (0.89–3.42)	0.316
No	13.0 (11.5–15.6)				
Yes	8.3 (7.7–11.3)				
Bone metastasis after treatment		1.33 (0.80–2.19)	0.273	1.43 (0.66–3.62)	0.450
No	13.0 (11.1–15.6)				
Yes	11.5 (9.7–16.0)				
Liver metastasis after treatment		1.65 (0.96–2.86)	0.078	2.19 (0.98–5.46)	0.082
No	13.0 (10.4–16.0)				
Yes	12.0 (8.8–13.7)				
Pleural effusion after treatment		1.35 (0.81–2.23)	0.249	1.55 (0.68–4.10)	0.379
No	13.0 (11.5–15.1)				
Yes	11.1 (9.8–15.6)				
New metastasis site		2.51 (1.52–4.11)	< 0.001	3.35 (1.27–8.82)	0.014
No	14.5 (13.0–18.2)				
Yes	9.0 (8.3–12.1)				
*EGFR* plasma after treatment		3.58 (1.77–7.25)	< 0.001	6.13 (1.79–21.01)	0.003
Negative	NR (13.7-NR)				
Positive	11.1 (9.5–12.9)				
Secondary T790M		1.94 (1.19–3.17)	0.008	3.01 (1.22–7.41)	0.017
Negative	13.8 (12.6–17.5)				
Positive	10.0 (8.8–12.1)				
cfDNA (ng/mL)		1.47 (0.88–2.46)	0.144	1.82 (0.80–4.74)	0.217
≤ 200	12.9 (11.0–19.5)				
> 200	12.2 (10.7–13.8)				

*Che* chemotherapy, *Ra* radiotherapy, *Sur* surgery, *NR* not reached

^a^ For the newly diagnosed group

**Fig. 2:**
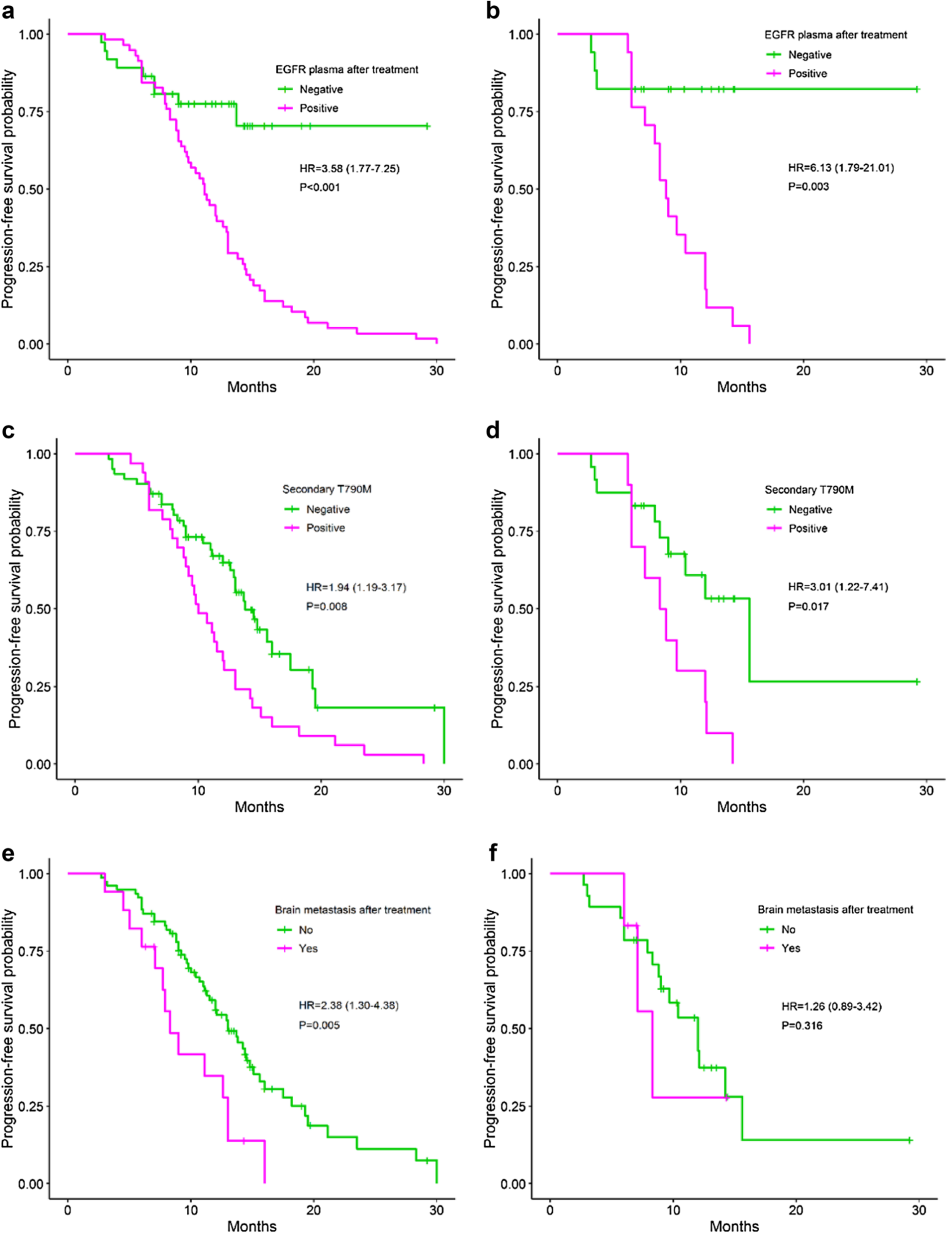
Progression-free survival between groups: *EGFR* plasma after treatment (**a**^£^, **b**^ǂ^); secondary T790M mutation (**c**^£^, **d**^ǂ^); brain metastasis after treatment (**e**^£^, **f**^ǂ^). £: all patients; ǂ: the newly diagnosed group. *EGFR* epidermal growth factor receptor

**Fig. 3:**
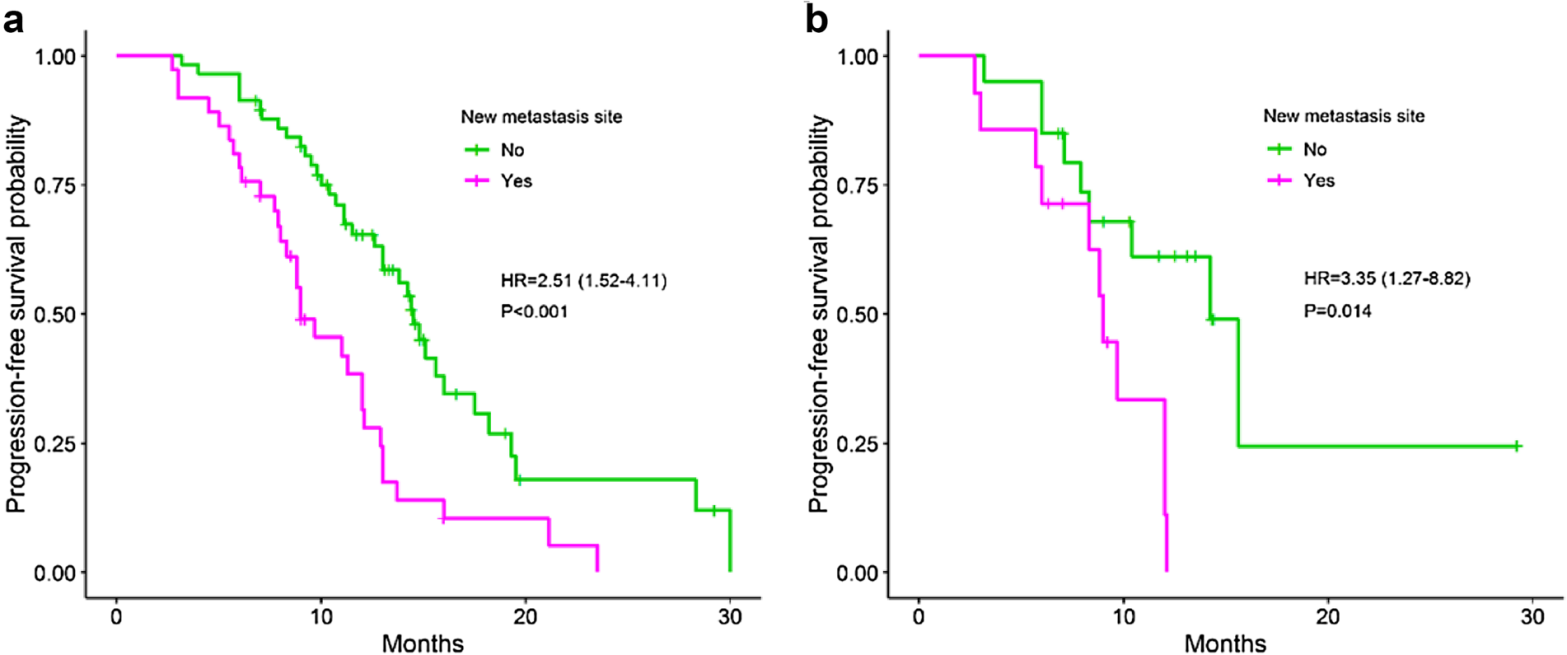
Progression-free survival between groups: new metastasis site (**a**^£^, **b**^ǂ^). £: all patients; ǂ: the newly diagnosed group

Univariate analysis has shown that patients with the occurrence of *EGFR* mutations in post-treatment plasma have the shorter PFS (11.1 months) as compared to *EGFR* negative patients (not reached), HR = 3.58, 95% CI 1.77–7.25 (*P *< 0.001) (Fig. 2a). Likewise, patients with secondary T790M mutation in plasma have the shorter PFS (10.0 months) compared to those of the negative group (13.8 months) (*P *= 0.008) (Fig. 2c). In addition, brain metastasis after treatment (Fig. 2e) and new metastasis site (Fig. 3a) are also two predictive factors for shortening of PFS in NSCLC patients treated with EGFR TKI, HR = 2.38 (*P *= 0.005), and 2.51 (*P *< 0.001), respectively. In further analyses with data of the newly diagnosed group, the similar results were obtained (Figs. 2b, d, f, 3b). Patients treated with TKI after surgery have the longer PFS (14.8 months) compared to those of TKI alone (12.1 months) or TKI and chemo-/radiotherapy (11.1 months), but not with statistically significant (*P *= 0.051). No differences in PFS between groups of other characteristics were observed (Table 2).

**Fig. 4:**
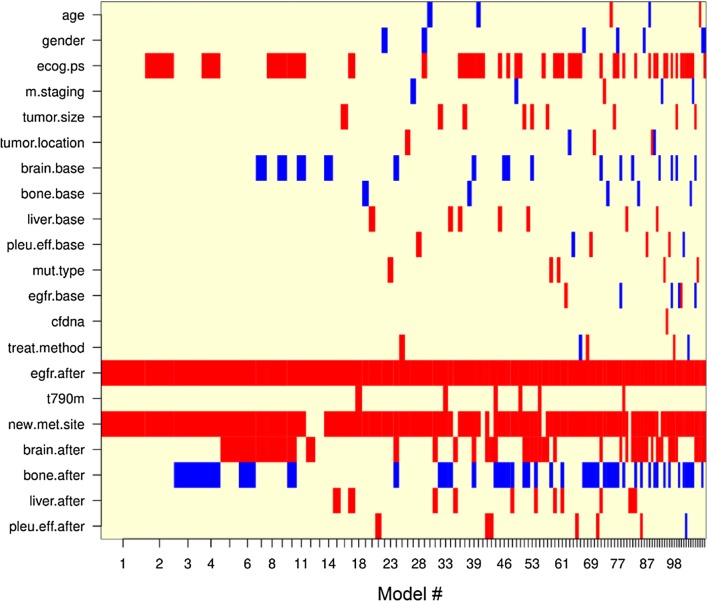
The possibility that factors appear in prognostic models for PFS by the Bayesian Model Averaging method. *EGFR* mutations in post-treatment plasma (egfr.after) and new metastasis (new.met.site) factors present in most models (100% and 95%, respectively)

Multivariate analysis has shown that *EGFR* mutation status in post-treatment plasma with brain and new metastasis at post-treatment are three independent predictive factors for resistance (*P *= 0.008, 0.016 and 0.028, respectively) (Table 3). Whereas, data of the newly diagnosed patients showed that only *EGFR* plasma test was identified as the independent factor, HR = 5.21 (*P *= 0.014). This might be due to the limited sample size of the group. The predictive power of *EGFR* plasma mutation test and new metastasis factor were confirmed by the BMA statistical analysis as factors in the best model for resistance (Bayesian information criterion value was − 15.8). The probability that the *EGFR* plasma test associated with the resistance risk was 100% (Fig. 4). This value of new metastasis factor was 95%.

**Table 3:** The independent predictive factors for progression-free survival

Characteristic	HR (95% CI)	*P*-value	HR^a^ (95% CI)	*P*-value^a^
*EGFR* plasma after treatment	3.53 (1.38–9.04)	0.008	5.21 (1.38–19.62)	0.014
Brain metastasis after treatment	2.87 (1.21–6.78)	0.016	1.55 (0.52–5.79)	0.512
New metastasis site	2.23 (1.09–4.57)	0.028	1.69 (0.71–4.73)	0.311

^a^ For the newly diagnosed group

### Overall survival between groups and prognostic factors

As of July 2018, 33 patients died after progression while 22 resistant cases were lost to follow-up. Eleven cases were continued with TKI treatment or changed to chemotherapy, radiotherapy. Median OS for all patients was 29.5, 95% CI 25.2–41.3 months. The median OS between groups of *EGFR* mutations and clinical characteristics were estimated and presented in Table 4.

**Table 4:** Univariate analysis for overall survival

Characteristic	OS, month (95% CI)	HR (95% CI)	*P*-value	HR^a^ (95% CI)	*P*-value^a^
Age, years		2.03 (0.99–4.14)	0.051	3.71 (1.00–13.79)	0.050
< 61	29.5 (28.6-NR)				
≥ 61	25.4 (18.7–41.3)				
Gender		0.88 (0.41–1.61)	0.555	0.74 (0.36–2.35)	0.616
Female	29.5 (25.2-NR)				
Male	29.5 (18.7-NR)				
ECOG PS		1.62 (0.66–3.99)	0.297	1.19 (0.36–5.47)	0.821
0–1	29.5 (25.2–41.3)				
≥ 2	26.4 (15.4-NR)				
Tumor location		0.91 (0.59–1.35)	0.588	1.21 (0.59–2.48)	0.599
Right lung	28.8 (22.3-NR)				
Left lung	29.5 (23.8-NR)				
Right + left	29.5 (17.6-NR)				
Tumor size at baseline (cm)		0.75 (0.47–1.14)	0.171	0.82 (0.42–1.21)	0.257
≤ 3	26.2 (11.5–29.5)				
> 3–5	26.7 (23.8-NR)				
> 5	29.5 (22.3-NR)				
M-Staging		1.72 (0.99–3.01)	0.056	1.04 (0.50–2.65)	0.942
M0	NR (16.0-NR)				
M1a	NR (25.2-NR)				
M1b	25.4 (21.8–39.1)				
Brain metastasis at baseline		1.12 (0.53–2.37)	0.759	0.79 (0.41–1.93)	0.371
No	29.5 (23.8-NR)				
Yes	28.6 (19.7–37.2)				
Bone metastasis at baseline		2.46 (1.22–4.94)	0.011	2.23 (0.91–7.06)	0.121
No	29.5 (25.8-NR)				
Yes	25.4 (18.7-NR)				
Liver metastasis at baseline		1.55 (0.71–3.36)	0.267	1.62 (0.44–6.01)	0.469
No	29.5 (25.2-NR)				
Yes	25.4 (18.7-NR)				
Pleural effusion at baseline		0.92 (0.44–1.93)	0.825	0.97 (0.45–3.27)	0.965
No	29.7 (23.8-NR)				
Yes	25.8 (23.8-NR)				
*EGFR* mutation type		1.50 (0.75–2.98)	0.247	1.26 (0.39–4.01)	0.693
E19del	28.9 (23.8-NR)				
L858R	25.8 (19.7–29.5)				
*EGFR* plasma at baseline		8.18 (2.06–32.49)	< 0.001	5.69 (1.48–21.95)	0.011
Negative	NR (21.3-NR)				
Positive	18.3 (15.0-NR)				
Unknown	29.5 (25.2-NR)				
Treatment method		1.69 (1.12–2.61)	0.016	2.61 (1.09–6.38)	0.033
TKI alone	25.2 (18.3-NR)				
TKI + Che/Ra	28.6 (18.7-NR)				
TKI + Sur	NR (25.8-NR)				
Brain metastasis after treatment		2.33 (1.08–5.05)	0.032	1.73 (0.66–6.45)	0.411
No	29.5 (25.2-NR)				
Yes	22.3 (8.9-NR)				
Bone metastasis after treatment		2.78 (1.40–5.54)	0.003	1.74 (0.53–5.77)	0.363
No	NR (25.8-NR)				
Yes	23.8 (18.3–29.5)				
Liver metastasis after treatment		2.01 (0.97–4.17)	0.059	1.14 (0.35–3.67)	0.831
No	34.9 (25.4-NR)				
Yes	23.8 (18.3-NR)				
Pleural effusion after treatment		1.16 (0.56–2.39)	0.686	0.71 (0.33–2.71)	0.488
No	29.4 (25.4-NR)				
Yes	25.2 (21.8-NR)				
New metastasis site		3.59 (1.76–7.33)	< 0.001	2.11 (0.98–6.69)	0.052
No	NR (25.8-NR)				
Yes	22.3 (18.3–28.6)				
*EGFR* plasma after treatment		2.66 (1.10–6.45)	0.031	3.37 (0.96–10.34)	0.059
Negative	NR (29.5-NR)				
Positive	25.2 (21.8–35.9)				
Secondary T790M		1.41 (0.70–2.83)	0.342	3.64 (1.02–12.96)	0.047
Negative	29.5 (25.2-NR)				
Positive	25.8 (17.6-NR)				
CfDNA (ng/mL)		2.34 (1.15–4.80)	0.019	6.27 (1.85–21.24)	0.003
≤ 200	NR (23.8-NR)				
> 200	25.8 (17.6-NR)				

*Che* chemotherapy, *Ra* radiotherapy, *Sur* surgery, *NR* not reached

^a^ For the newly diagnosed group

**Fig. 5:**
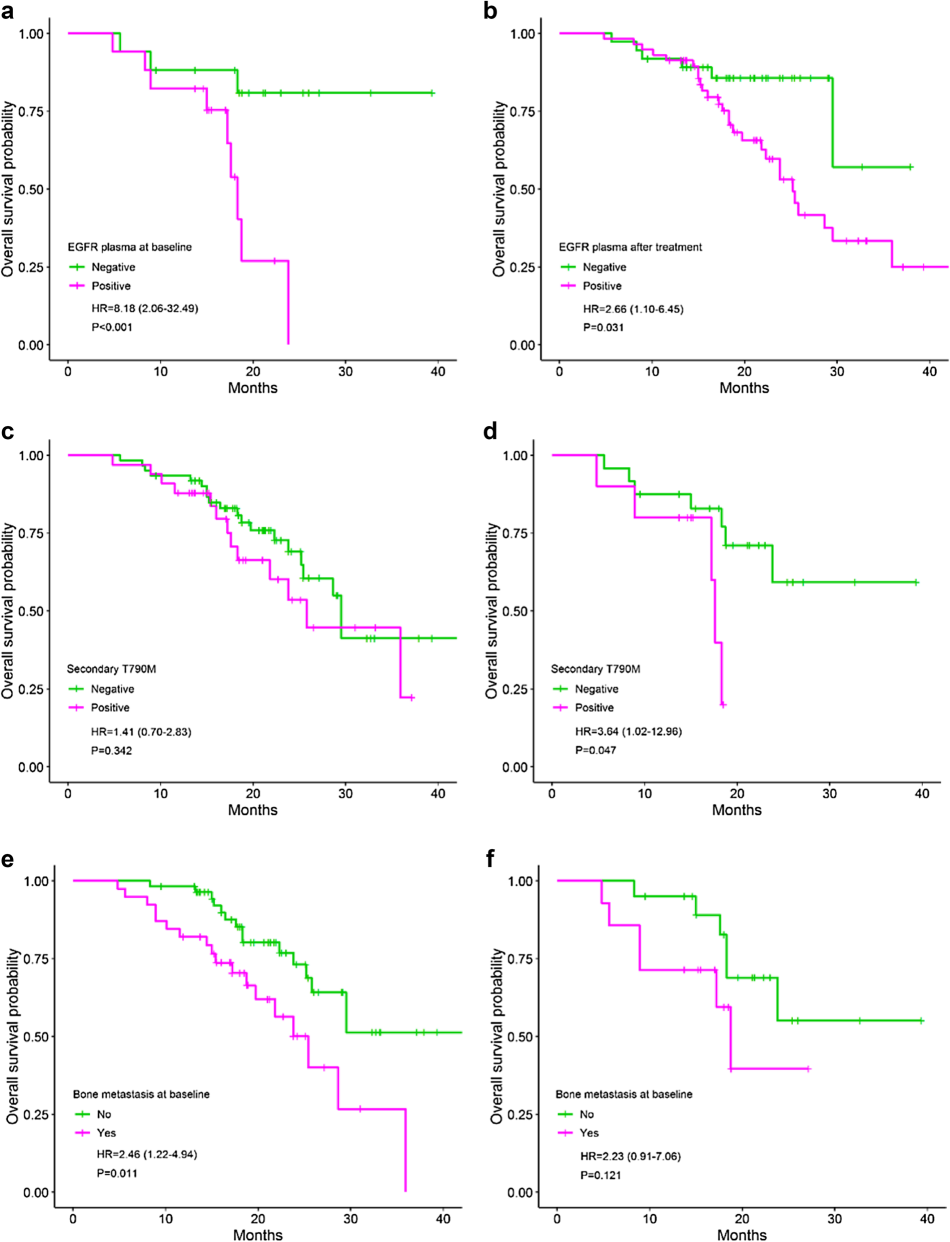
Overall survival between groups: *EGFR* plasma at baseline (**a**); *EGFR* plasma after treatment (**b**); secondary T790M mutation (**c**^£^, **d**^ǂ^); bone metastasis at baseline (**e**^£^, **f**^ǂ^). £: all patients; ǂ: the newly diagnosed group. *EGFR* epidermal growth factor receptor

**Fig. 6:**
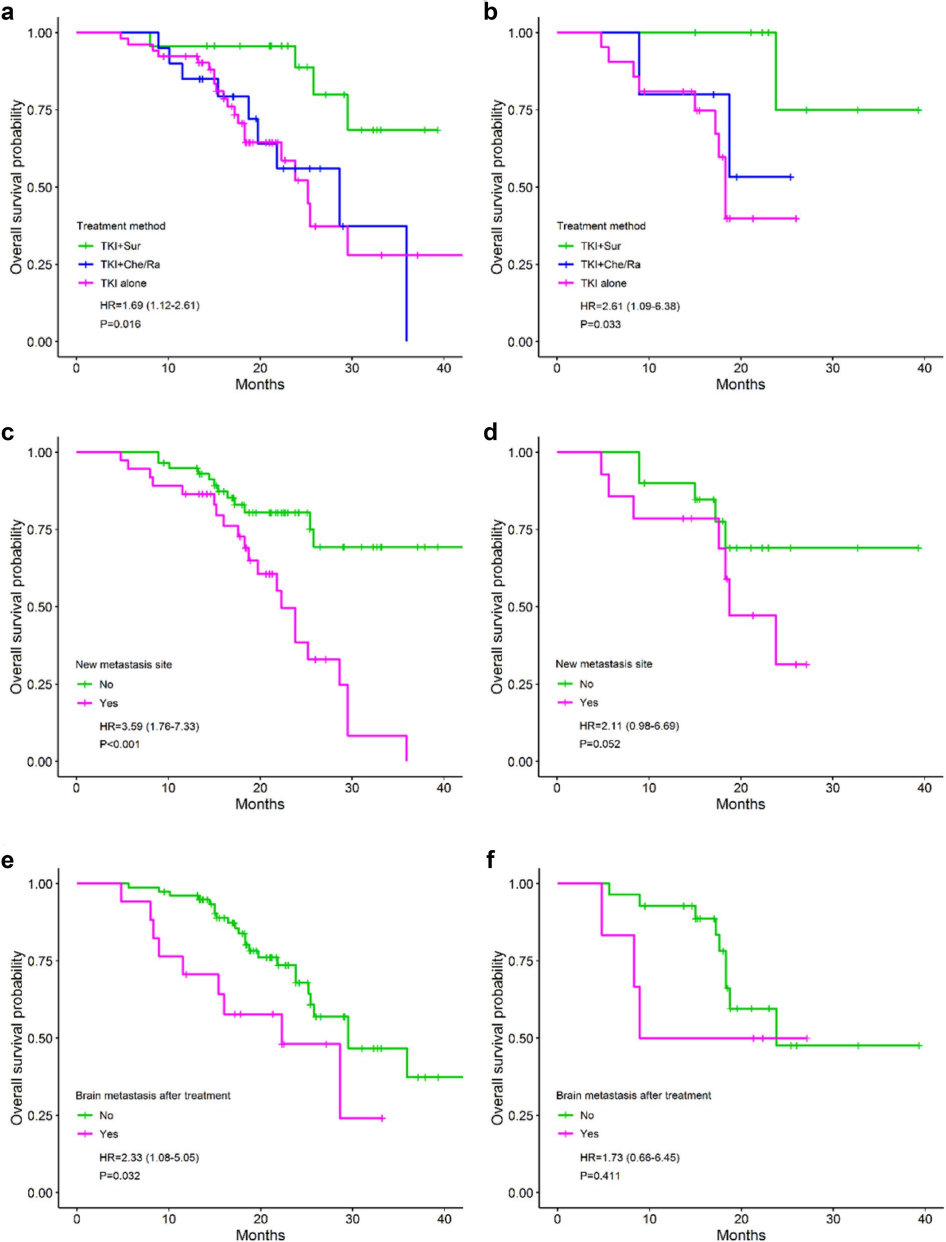
Overall survival between groups: treatment method (**a**^£^, **b**^ǂ^); new metastasis site (**c**^£^, **d**^ǂ^); brain metastasis after treatment (**e**^£^, **f**^ǂ^). £: all patients; ǂ: the newly diagnosed group. *TKI* tyrosine kinase inhibitor, *Sur* surgery, *Che* chemotherapy, *Ra* radiotherapy

**Fig. 7:**
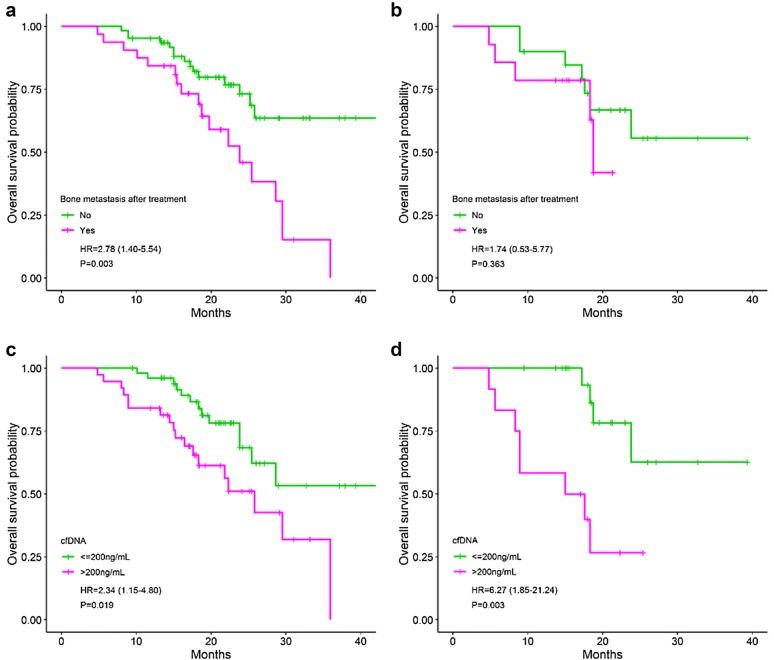
Overall survival between groups: bone metastasis after treatment (**a**^£^, **b**^ǂ^); and cfDNA level (**c**^£^, **d**^ǂ^). £: all patients; ǂ: the newly diagnosed group

By the univariate analyses, patients with *EGFR* positive plasma at baseline and post-treatment have the shorter OS (18.3 and 25.2 months, respectively) compared to those of negative group (not reached), HR = 8.18 (*P *< 0.001) and 2.66 (*P *= 0.031), respectively (Fig. 5a, b). Notwithstanding, the role of plasma-based secondary T790M mutation in prognosis for OS was not clear (25.8 months in positive versus 29.5 months in negative patients) (Fig. 5c, d). Patients who have the distant metastases to the bone at baseline (*P *= 0.011, Fig. 5e) and post-treatment (*P *= 0.003, Fig. 7a), the new lesion (*P *< 0.001, Fig. 6c) or brain metastasis at post-treatment (*P *= 0.032, Fig. 6e) have the shorter OS compared to others. Otherwise, surgery before TKI treatment help to prolong the OS time in NSCLC (not reached versus 28.6 months in TKI + chemo-/radiotherapy and 25.2 months in TKI alone) (*P *= 0.016). This difference was observed in all patients as well as in the newly diagnosed group (Fig. 6a, b). The high level of cfDNA concentration was closely associated with the worse survival (*P *= 0.019) which was regenerated by the limited data of the newly diagnosed group (*P *= 0.003) (Fig. 7c, d). Liver metastasis after treatment also was an inferior prognostic factor for OS (23.8 versus 34.9 months) but not with statistically significant (*P *= 0.059).

**Fig. 8:**
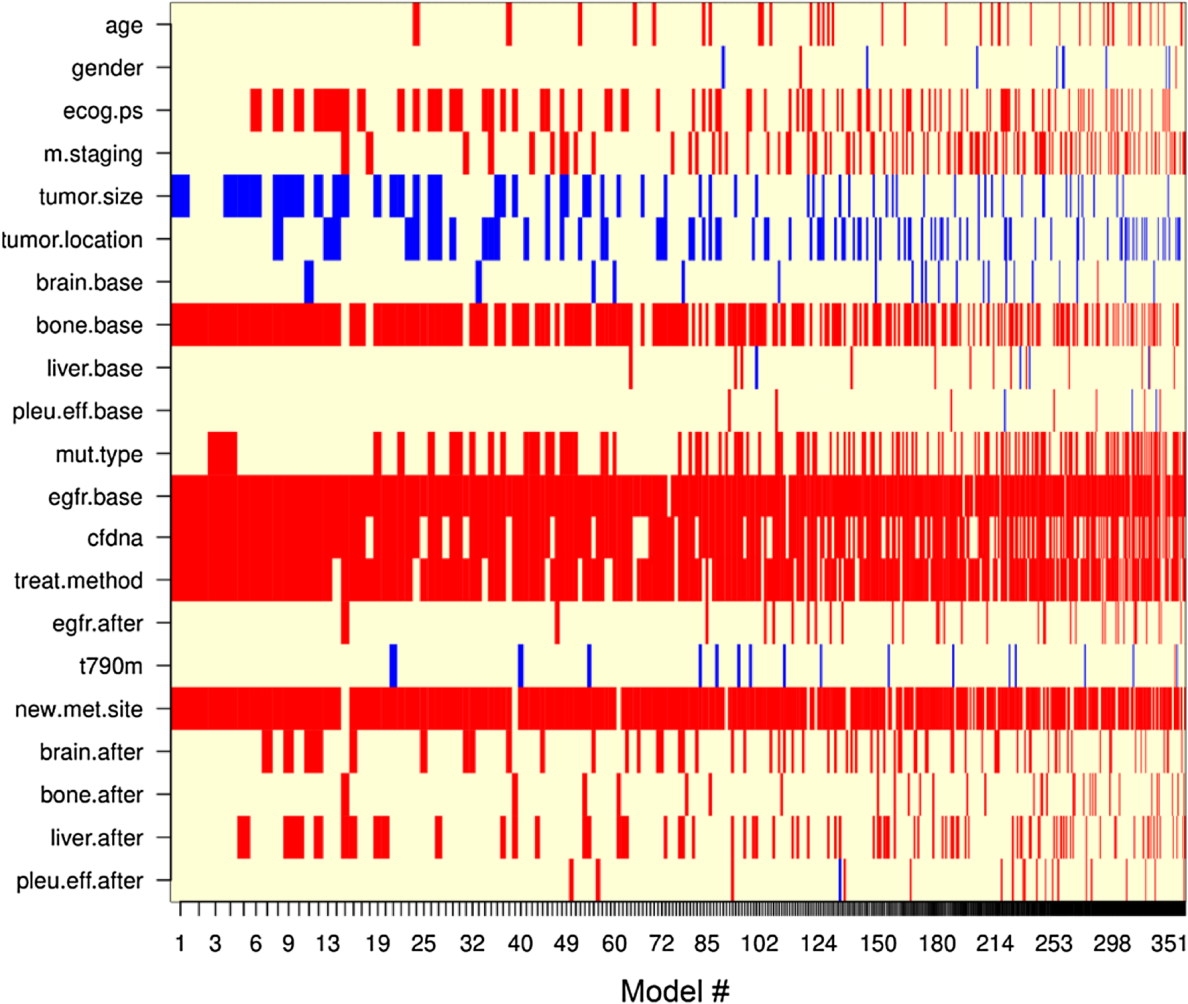
The possibility that factors appear in prognostic models for OS by the Bayesian Model Averaging method. Bone metastasis at baseline (bone.base: 68.1%); *EGFR* plasma at baseline (egfr.base: 99.7%); cfDNA (cfdna: 73.4%); treatment method (treat.method: 83.5%); and new metastasis (new.met.site: 93.2%) factors present in most models

In the multivariate analysis, five factors were recognized as the independent prognostic factors for OS including *EGFR* plasma and bone metastasis at baseline, treatment method, new metastasis site and cfDNA (*P *= 0.044, 0.012, 0.012, 0.003 and 0.004, respectively) (Table 5). The BMA analysis demonstrated that the detected *EGFR* mutations in baseline plasma and new lesion factor (probability of 99.7% and 93.2%, respectively) are strongly linked to the poor survival (Fig. 8). The possibility that treatment method, cfDNA, and bone metastasis at baseline affect the survival are 83.5%, 73.4%, and 68.1%, respectively.

**Table 5:** The independent predictive factors for overall survival

Characteristic	HR (95% CI)	*P*-value	HR^a^ (95% CI)	*P*-value^a^
*EGFR* plasma at baseline	3.65 (1.04–12.87)	0.044	6.87 (1.13–41.66)	0.036
Bone metastasis at baseline	2.73 (1.25–5.98)	0.012	14.92 (1.88–96.63)	0.010
Treatment method	1.97 (1.16–3.35)	0.012	5.48 (1.12–26.77)	0.035
New metastasis site	2.99 (1.44–6.21)	0.003	4.63 (1.22–17.61)	0.034
cfDNA	3.23 (1.45–7.20)	0.004	29.67 (3.98–91.36)	< 0.001

^a^ For the newly diagnosed group

## Discussion

To date, although plasma sample cannot replace the tumor tissue in *EGFR* mutation testing, it contains cfDNA derived from different tumor locations, and thus seem to be more effective than tissue in reflecting the gene alterations during targeted treatment. This is very helpful for further clinical decisions.

In this study, we monitored *EGFR* mutations in serial plasma samples of Adenocarcinoma patients treated with EGFR TKI and shown a negative result of patients who still archived partial response or stable disease. In conversely, most resistant patients (86.4%) were positive for *EGFR* mutations. Besides the initial mutation type as E19del or L858R only, almost half of the resistant patients (48.5%) carried the secondary T790M mutation in plasma sample which is consistent with results of previous studies (21–53%) [8–19, 21, 31]. This indicates that *EGFR*, especially T790M mutation can be detected with high frequency in plasma by scorpions ARMS method, which is the basis for switching to the subsequent treatment such as Osimertinib for patients. Interestingly, one-fourth of resistant patients have the recurrence of *EGFR* mutations prior to progression 2.1 months (from 0.6 up to 7.9 months). This was just demonstrated in a few studies before [11, 14, 15, 21]. In clinical practice, the rapidly changing to the new treatment methods once detected *EGFR* mutations early is very important and beneficial, especially for old patients or whom with severe symptoms.

The previous clinically-experimental studies have shown that the maintenance of positive status of *EGFR* mutations in plasma after 4 weeks to 3 months of TKI treatment is the hallmark of early resistance [13, 21, 28, 29]. In the present work, we recorded that four factors, including *EGFR* mutations, T790M mutation, brain, and new metastasis after treatment are prognostic factors for worse PFS. The association of positive status of *EGFR* in post-treatment plasma with inferior PFS is consistent with previous findings [13, 17, 21, 28, 29]. When assessing the role of *EGFR* plasma test and T790M mutation in correlation with clinical characteristics at prior and after TKI treatment as confounders, we did note that not T790M mutation, *EGFR* mutations in general with new metastasis and brain metastasis factors are independent prognostic factors for the shortening of PFS or early resistance. Of which, *EGFR* mutations and new metastasis factor which is a criterion of the RECIST evaluation standard contributed to the best model for resistance. This finding is different from the result of the previous study [19]. We assume that the presence of *EGFR* plasma test as a covariate in the prediction model effects on the possibility that T790M mutation is an independent prognostic factor or not. It is easy to see that secondary T790M mutation often appears together with the E19del or L858R mutation. Besides, the prognostic models might be affected by the presence of more clinical characteristics. The probability of 100% that *EGFR* plasma test associated with the resistance, suggesting that this factor might be more important than new metastasis factor (possibility of 95%) and other RECIST criteria in predicting the drug resistance.

In this study, four worse prognostic factors (pre-treatment *EGFR* plasma and bone metastasis, new lesion, and high cfDNA concentration) with one favorable factor (surgery) were identified as the best model for overall survival. All factors in this model were significant in a small sample size of the newly diagnosed group as well. The positive status of *EGFR* plasma at pre- and post-treatment as the worse factor is in accordance with previous studies [14, 15, 21, 28]. Notably, the baseline *EGFR* plasma plays the role as the most powerful factor among five independent factors for survival (possibility of 99.7%). This is consistent with the study of Kim et al. [21] (baseline *EGFR* plasma plays the role as the independent factor for worse OS), but different with two other studies (T790M mutation is the independent factor for OS) [15, 19]. A similar point between these two studies is that *EGFR* plasma, in general, was not considered as a covariate in prognostic models which is different with our study. Whereas, in current work, we use the BMA statistical analysis to identify the significant factors (Zheng et al. used the backward stepwise selection process by Akaike information criterion value). These might be the causes of different results between our study and two above studies.

This study evaluates the prognostic role of *EGFR* plasma test in correlation with clinical factors at pre- and post-treatment, however, has limitations of a single center study. Smoking history was not sufficient for all study subjects, so that has not been a co-variable in prognostic models. This has been proposed by previous studies for considering in TKI treatment evaluation [32]. A further research which includes clinical factors with smoking variable should be conducted for assessing the prognostic role of *EGFR* plasma test in NSCLC.

In conclusion, the negative status of *EGFR* mutations in plasma of post-TKI treatment may help to predict a good achievement. Otherwise, the early presenting of *EGFR* mutations, especially the secondary T790M mutation in plasma help to predict the drug resistance. *EGFR* plasma test is the powerfully prognostic factor for worse survival in NSCLC treated with EGFR TKI regardless of clinical characteristics.

## Abbreviations

EGFR TKI: epidermal growth factor receptor tyrosine kinase inhibitor
NSCLC: non-small cell lung cancer
OS: overall survival
PD: progressive disease
PFS: progression-free survival
PR: partial response
SD: stable disease
NR: not reached
